# HIV serology false positivity among expatriates from Africa: a screening dilemma

**DOI:** 10.1099/jmm.0.001186

**Published:** 2020-05-29

**Authors:** Hanan Al-Kindi, Amina Al-Jardani

**Affiliations:** ^1^​ Central Public Health Laboratories, Ministry of Health, Muscat, Oman

**Keywords:** HIV serodiagnosis, false-positive reaction, HIV core protein p24, HIV-1, immunoassay, routine diagnostic tests

## Abstract

HIV prevalence in Oman is low (<5 %); however, 45 % of the population are expatriates, including a portion originating from countries with high HIV prevalence (>5 %). HIV screening is performed at regional public health laboratories as part of a medical fitness programme for residency applicants. We conducted a retrospective evaluation of indeterminate serology results from 11 females of African origin, aged 21–43 years. Serology testing for HIV was conducted according to the national Oman algorithm: fourth-generation immunoassays (Bio-Rad GS HIV Combo Ag/Ab EIA, Siemens Enzygnost HIV Integral 4, Abbott ARCHITECT HIV Ag/Ab Combo, Roche Elecsys HIV Combi PT, bioMérieux VIDAS HIV DUO QUICK), confirmatory assays (Geenius HIV 1/2 Confirmatory, INNO-LIA HIV I/II Score) and PCR testing. Confirmatory testing to resolve indeterminate results was conducted with available samples for five patients using a combination of immunoassays, confirmatory assays, PCR/PERT and pro-viral DNA levels, at three external laboratories; Roche Diagnostics (Germany), Swiss National Laboratory (Switzerland) and Barts Health NHS Trust (UK). Nineteen serum, 15 plasma and two whole-blood samples were analysed. Nine of ten patients analysed on Bio-Rad and Siemens immunoassays were highly reactive; seven were highly reactive on the Abbott assay. Eight of nine patients tested with the Roche assay were negative. Three of four patients tested on the bioMérieux assay were negative. Five patients underwent confirmatory testing at external laboratories; all were negative by HIV-RNA or pro-viral DNA testing. In conclusion, HIV-RNA and pro-viral DNA testing is recommended for HIV screening of individuals from high-prevalence regions coming to low-prevalence regions.

## Introduction

The human immunodeficiency virus (HIV) pandemic continues to present a global health challenge to clinicians and diagnostic laboratories. Prevalence of HIV varies between countries and, according to the World Health Organization (WHO), prevalence among the population of Oman is low (<5 %) [1, 2]. However, expatriates account for 45 % of the general population of Oman, and a portion of visa applicants originate from countries with high prevalence of HIV (>5 %) [1].

To prevent the spread of HIV in Oman, individuals applying for residency or work are routinely screened before visa approval. Diagnostic HIV testing is also offered to all residents with HIV-related symptoms, as an early diagnosis of HIV can improve patient management. Additionally, routine HIV testing is offered to asymptomatic residents at risk of HIV infection (e.g. pregnant women and patients with sexually transmitted infections, tuberculosis or hepatitis B/C). A national HIV testing algorithm is followed to ensure diagnostic standardization, involving consecutive testing with two fourth-generation HIV immunoassays followed by confirmatory testing with an antibody differentiation immunoassay (ADI), a Western blot (WB)-like line immunoassay and PCR testing, if required. This procedure is consistent with international guidelines from the Centers for Disease Control (CDC) and WHO [3–5].

The aim of this study was to evaluate the potential diagnostic challenges encountered with routine HIV testing among expatriates from Africa. We report 11 cases of African individuals undergoing routine HIV screening during application for residency in Oman, for whom indeterminate serology (i.e. no clear HIV-positive or HIV-negative result) was obtained. Confirmatory testing at reference laboratories abroad demonstrated that results that were initially reactive with some fourth-generation HIV immunoassays were false-positives.

### Study design

This was a retrospective report of cases that occurred during routine HIV screening in Oman. HIV screening was undertaken at regional public health laboratories as part of a medical fitness programme for all residents. Serum samples were collected from patients at private institutions and regional public health laboratories. Verbal informed consent was acquired from each patient before testing, alongside relevant patient information and contact details. All patients received pre-test information and voluntary post-test counselling; issues discussed between counsellor/health care provider and patients were confidential and not disclosed to a third party without prior consent.

HIV screening was conducted according to the national algorithm [1]. Patient serum samples that were reactive when evaluated using a first fourth-generation immunoassay (A1) were sent for confirmatory testing with a second fourth-generation immunoassay (A2) at a central public health laboratory (CPHL). A cut-off index (COI) of >1.0 was used to determine reactivity. If both A1 and A2 were reactive, an ADI was used to confirm HIV infection and differentiate HIV-1 from HIV-2. If ADI testing was indeterminate, plasma HIV-RNA levels were determined using PCR and a WB-like line immunoassay. HIV-positive status was only disclosed after confirmation of infection at a CPHL and repeat HIV serology in a re-bleed sample.

Choice of serology test (A1 and A2) for initial screening depended on platforms available at each screening site. Following unusually high-level reactivity according to GS HIV Combo Ag/Ab EIA (Bio-Rad Laboratories, USA; manual and automated systems), Enzygnost HIV Integral 4 (Siemens, Germany), and ARCHTECT HIV Ag/Ab Combo (Abbott Laboratories, USA) assays, some cases were further investigated using the Elecsys HIV combi PT assay (Roche Diagnostics, Switzerland; cobas e 411 analyser) or VIDAS HIV DUO QUICK assay (bioMérieux, France). For confirmatory testing, an ADI assay (Geenius HIV 1/2 Confirmatory [Bio-Rad Laboratories, USA]) and/or WB-like line immunoassay (INNO-LIA HIV I/II Score [Innogenetics NV, Belgium]) were used. PCR testing was done either on the first sample or a repeated sample, according to the national algorithm, using the cobas HIV-1 test (cobas 4800 analyser).

Where available, samples were also sent to three external laboratories (Roche Diagnostics, Penzberg, Germany; Swiss National Laboratory, Zurich, Switzerland; Barts Health NHS Trust, London, UK) for confirmatory testing. Analyses at the UK laboratory used VIDAS HIV DUO QUICK, Geenius HIV 1/2 Confirmatory and INNO-LIA HIV I/II Score assays and confirmatory PCR and pro-viral DNA testing. Pro-viral DNA was not performed for all patients, as it requires an unseparated whole-blood EDTA sample, collected and transported to the reference laboratory within 5–7 days of collection. Analyses in Germany and Switzerland used VIDAS HIV DUO QUICK and Elecsys HIV Combi PT (cobas e 601 analyser) assays, followed by confirmatory INNO-LIA HIV I/II Score assay and testing using a product-enhanced reverse transcriptase (PERT) assay (in-house test accredited for human plasma; Swiss National Center for Retroviruses, Institute of Medical Virology – University of Zurich, Switzerland).

All assays and tests were performed according to manufacturer recommended protocols. In accordance with national guidelines, all screening assays and tests were carried out in public health laboratories, the confirmatory testing was performed by the CPHL and quality-assurance processes (independent testing) were followed to ensure correct diagnoses in the case of discrepant results.

## Results

Serum samples were collected from 11 females of African origin, aged 21–43 years, between November 2016 and January 2018. A total of 19 serum, 15 plasma and two whole-blood samples were analysed. Four patients (no. 1, 2, 3 and 4) had two samples tested >7 days apart at CPHL and two patients (no. 5 and 6) had one sample tested in Oman. Four patients (no. 7, 8, 9 and 10) had their initial sample tested at CPHL and a follow-up sample tested at Barts Health NHS Trust. One patient (no. 11) had a sample tested at Roche Diagnostics/University of Zurich.

**Fig. 1.**
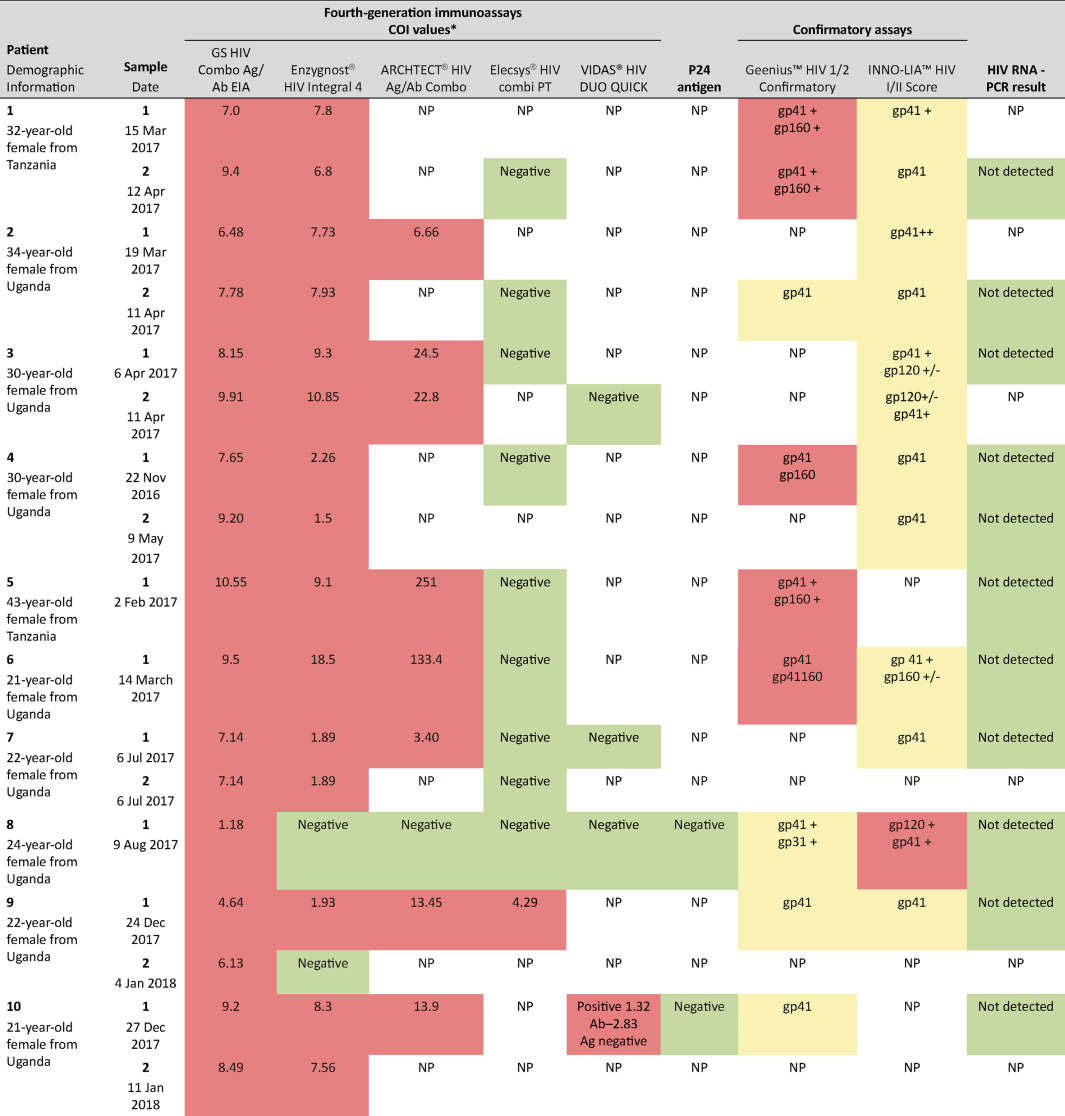
Summary of HIV screening and confirmatory testing results performed in Oman. Colours are as follows: red=positive; yellow=indeterminate; green=negative. COI, cut-off index; NP, not performed. *COI>1.0=reactive, for all immunoassays.

Indeterminate serological marker results were observed for all samples when tested by multiple fourth-generation HIV assays in Oman (Fig. 1). Seventeen serum samples from the first ten patients were analysed with both GS HIV Combo Ag/Ab EIA and Enzygnost HIV Integral 4 assays; all except patient 8 had high-level reactivity (COIs: 4.64–10.55; 1.50–18.5, respectively). High-level reactivity was also detected using the ARCHITECT HIV Ag/Ab Combo assay on eight samples from seven patients (COIs: 3.40–251). Four samples from four patients were tested retrospectively using the VIDAS HIV DUO QUICK assay; one sample was reactive, with negative antigen components. Ten samples from nine patients were tested with the Elecsys HIV combi PT assay in Oman; all were negative except for patient 9, who had low-level reactivity (COI: 4.29). Confirmatory testing with the Geenius HIV 1/2 assay was performed on nine samples from eight patients: four had indeterminate results (three with only gp41 detected; one with gp41 and gp31 detected), and four tested positive for HIV-1 with weak gp160 and strong gp41 detected. Analysis of 12 samples from eight patients using the INNO-LIA HIV I/II Score assay gave indeterminate results for all patients, based on a gp41 score of 2 or 3+, with or without a gp120±score. HIV-RNA was not detected by PCR in any sample from the 11 patients.

**Fig. 2.**
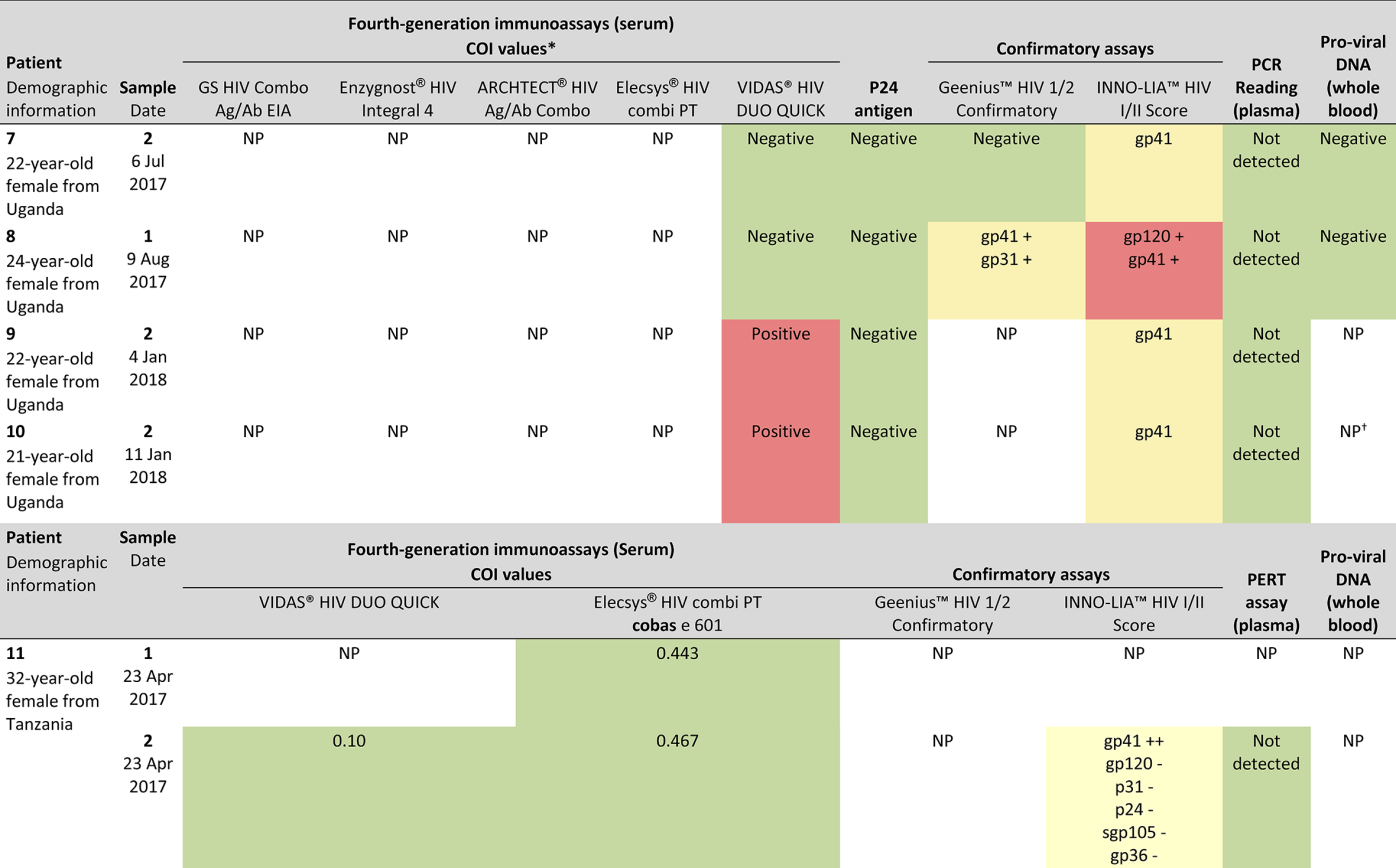
Results of HIV confirmatory testing performed at UK, Swiss and German laboratories. Colours are as follows: red=positive; yellow=indeterminate; green=negative. COI, cut-off index; NP, not performed; PERT, product-enhanced reverse transcriptase. *COI>1.0=reactive, for all immunoassays. †Whole blood was not sent to UK, Swiss and German laboratories for this patient.

To resolve the indeterminate results from initial screening, six samples from five patients were sent for external confirmatory testing; all were determined to be HIV negative (Fig. 2).

Of four samples from four patients retested at Barts Health NHS Trust, two had negative serology and were indeterminate. HIV-RNA was not detectable in any sample; pro-viral DNA testing was also negative for patients 7 and 8. All four patients were confirmed HIV negative.

Two samples from one patient were retested by Roche Diagnostics and the Swiss National Laboratory (Fig. 2). COIs of measurements were 0.10 using VIDAS HIV DUO QUICK and 0.443–0.467 using Elecsys HIV Combi PT assays. Confirmatory testing with the INNO-LIA HIV I/II Score assay gave an indeterminate result for the re-bleed sample. With no evidence of HIV infection in this sample using PERT testing, the patient was confirmed to be HIV negative.

## Discussion

We report 11 cases where individuals of African origin tested false-positive for HIV infection based on multiple fourth-generation assays and confirmatory differentiation testing, according to Omani national guidelines [1]. The Elecsys HIV combi PT assay and PCR testing for HIV-RNA consistently determined all samples to be non-reactive/negative. Retesting by laboratories in Germany, Switzerland and the UK subsequently confirmed that all samples were negative for HIV infection.

Among the potential causes of these false-positive results, it is possible that some individuals may have been ‘elite controllers’, i.e. their immune systems were capable of suppressing viral replication without the need for antiretroviral therapy, resulting in undetectable levels of viral RNA in the blood [6]. However, this condition is very rare (<1 %) [7], and most cases are not confirmed by serology as HIV-1. Cross-reactivity between HIV-1 peptides and immunological factors (e.g. *Schistosoma* antibodies) has previously shown a strong association with false-positive HIV test results [8]. However, use of a PERT assay in one case was able to exclude the presence of other retroviral infections. Furthermore, previous exposure to HIV vaccination can result in strong reactivity during HIV screening, with no HIV-RNA detected [9]. It was not possible to account for previous exposure to HIV vaccination in the present study due to a lack of clinical history available for the patients that were tested.

All patients had very strong reactivity to an antigen that cross-reacts with HIV gp41 antibodies. Reactivity was also detected with gp160, but with lower intensity; this can likely be attributed to the gp41 component of this polyprotein, warranting further research into the nature of this antigen.

High false-positive rates are well documented for fourth-generation HIV immunoassays, particularly in areas of low HIV prevalence. Indeed, Kim *et al.* [10] reported a positive predictive value (PPV) of only 31.21 % for the ARCHITECT HIV Ag/Ab Combo assay when used alone, which may be attributed to heterophilic antibody interference [11, 12]. However, HIV-screening algorithms involving a confirmatory fourth-generation assay can achieve a high PPV (97 %) with these assays, endorsing the HIV-screening algorithm used in Oman [13].

High sensitivity (>95 %) and specificity (>99 %) have been observed with commercially available fourth-generation HIV assays, confirming their superior analytical performance compared with previous generations [14, 15]. The Geenius HIV 1/2 Confirmatory assay also shows high sensitivity (99.7 %) and specificity (98.4 %) [16]. Despite excellent sensitivity and specificity, low PPV rates for HIV screening assays remain problematic in regions of low HIV prevalence [10]. The present findings are reflective of this, with Bio-Rad, Siemens and Abbott assays providing false-positive serology readings in 10/10, 9/10 and 7/8 tested patients, respectively.

According to current Omani guidelines, a positive HIV-1 immunoassay and ADI are sufficient laboratory evidence for HIV-1 infection [1]. Furthermore, WHO guidelines for regions with a high prevalence of HIV consider two positive immunoassays as proof of HIV infection [5]. Our results indicate a need to include confirmatory pro-viral DNA testing in routine HIV screening, as all patient samples tested negative for pro-viral DNA, despite positive serology results with fourth-generation immunoassays. Pro-viral DNA testing can aid confirmation of HIV infection and identify elite controllers, which could inform clinical practice in regions with low HIV prevalence [6]. Clinicians and laboratory personnel should also consider the impact of potential cross-reactivity and ensure that HIV-RNA status is determined to decrease the risk of false-positive HIV diagnosis. This research does have some limitations. This was a retrospective case report; therefore, no pre-determined study protocol was followed. As such, it was not possible to standardize the type of sample tested (serum, plasma or whole blood) or the external laboratory used for confirmatory testing (Roche Diagnostics [Germany], Swiss National Laboratory [Switzerland], or Barts Health NHS Trust [UK]). However, HIV screening was conducted according to a national algorithm, which is consistent with international guidelines from the CDC and WHO, and reflective of real-world clinical practice in Oman [3–5]. Moreover, it is important to note that the gender of cases reported was likely biased due to local culture and available job opportunities for expatriates from African countries. No clinical history was available for any of the patients involved; therefore, it was not possible to assess risk factors for HIV infection. Furthermore, it was not possible to test for other causes of false-positive HIV serology such as schistosomiasis, malaria or Epstein-Barr virus [8]. In addition, generalizability of the present findings may be restricted due to the small number of single-sex cases reported. The small sample size also precludes any meaningful evaluation of a possible relationship between false-positive results and an individual antigen(s), such as HIV p24. Further studies are required to better explore these points.

Our findings suggest that HIV screening for individuals who originate from regions with high HIV prevalence may need to incorporate assessment of HIV-RNA status and confirmatory pro-viral DNA testing when performed in areas of low HIV prevalence. As a minimum, the rate of false-positive results among fourth-generation HIV immunoassays should be reviewed within the context of national guidelines to ensure that those with the lowest rates are selected for use in clinical practice. Further studies should investigate antibodies potentially responsible for these false-positive results.

## References

[1] Ministry of Health (2015). HIV management in Oman. A guide for health care workers.

[2] World Health Organization (2018). Prevalence of HIV among adults aged 15 to 49: estimates by country. http://apps.who.int/gho/data/view.main.22500?lang=en.

[3] Centers for Disease Control and Prevention (2014). Laboratory testing for the diagnosis of HIV infection: updated recommendations. https://stacks.cdc.gov/view/cdc/22423/cdc_22423_DS2.pdf.

[4] World Health Organization (2012). Service Delivery Approaches to HIV Testing and Counselling (HTC): A Strategic Policy Framework.

[5] World Health Organization (2015). Consolidated Guidelines on HIV Testing Services: 5Cs: Consent, Confidentiality, Counselling, Correct Results and Connection 2015.

[6] Joshi RP, Gomez CA, Steiner D, Aziz N, Pinsky BA (2017). The brief case: confirmed positive HIV-1 serologic screening but undetectable RNA virus load in a pregnant woman. J Clin Microbiol.

[7] Okulicz JF, Lambotte O (2011). Epidemiology and clinical characteristics of elite controllers. Curr Opin HIV AIDS.

[8] Everett DB, Baisely KJ, McNerney R, Hambleton I, Chirwa T (2010). Association of schistosomiasis with false-positive HIV test results in an African adolescent population. J Clin Microbiol.

[9] Voronin Y, Zinszner H, Karg C, Brooks K, Coombs R (2015). HIV vaccine-induced sero-reactivity: a challenge for trial participants, researchers, and physicians. Vaccine.

[10] Kim S, Lee J-H, Choi JY, Kim JM, Kim H-S (2010). False-positive rate of a "fourth-generation" HIV antigen/antibody combination assay in an area of low HIV prevalence. Clin Vaccine Immunol.

[11] Lang R, Charlton C, Beckthold B, Kadivar K, Lavoie S (2017). HIV misdiagnosis: a root cause analysis leading to improvements in HIV diagnosis and patient care. J Clin Virol.

[12] Lavoie S, Caswell D, Gill MJ, Kadkhoda K, Charlton CL (2018). Heterophilic interference in specimens yielding false-reactive results on the Abbott 4th generation architect HIV Ag/Ab Combo assay. J Clin Virol.

[13] Avidor B, Chemtob D, Turner D, Zeldis I, Girshengorn S (2018). Evaluation of the virtues and pitfalls in an HIV screening algorithm based on two fourth generation assays - A step towards an improved national algorithm. J Clin Virol.

[14] Delaney KP, Branson BM, Uniyal A, Phillips S, Candal D (2011). Evaluation of the performance characteristics of 6 rapid HIV antibody tests. Clin Infect Dis.

[15] Mitchell EO, Stewart G, Bajzik O, Ferret M, Bentsen C (2013). Performance comparison of the 4th generation Bio-Rad laboratories Gs HIV Combo Ag/Ab EIA on the EVOLIS automated system versus Abbott ARCHITECT HIV Ag/Ab Combo, Ortho anti-HIV 1+2 EIA on Vitros ECi and Siemens HIV-1/O/2 enhanced on Advia Centaur. J Clin Virol.

[16] Kosack CS, Page A-L, Beelaert G, Benson T, Savane A (2017). Towards more accurate HIV testing in sub-Saharan Africa: a multi-site evaluation of HIV RDTs and risk factors for false positives. J Int AIDS Soc.

